# Ceramide-Graphene Oxide Nanoparticles Enhance Cytotoxicity and Decrease HCC Xenograft Development: A Novel Approach for Targeted Cancer Therapy

**DOI:** 10.3389/fphar.2019.00069

**Published:** 2019-02-08

**Authors:** Shi-Bing Wang, Ying-Yu Ma, Xiao-Yi Chen, Yuan-Yuan Zhao, Xiao-Zhou Mou

**Affiliations:** ^1^Key Laboratory of Tumor Molecular Diagnosis and Individualized Medicine of Zhejiang Province, Zhejiang Provincial People’s Hospital, People’s Hospital of Hangzhou Medical College, Hangzhou, China; ^2^Clinical Research Institute, Zhejiang Provincial People’s Hospital, People’s Hospital of Hangzhou Medical College, Hangzhou, China

**Keywords:** hepatocellular carcinoma, graphene oxide, ceramide, apoptosis, drug-resistant

## Abstract

Despite substantial efforts to develop novel therapeutic strategies for treating hepatocellular carcinoma (HCC), the effectiveness and specificity of available drugs still require further improvement. Previous work has shown that exogenous ceramide can play a key role in inducing the apoptotic death of cancer cells, however, the poor water-solubility of this compound has hampered its use for cancer treatment. In the present study, we used polyethylene glycol (PEG) and polyethylenimine (PEI) co-conjugated ultra-small nano-GO (NGO-PEG-PEI) loaded with C6-ceramide (NGO-PEG-PEI/Cer) as a strategy for HCC treatment. We assessed the biological role of NGO-PEG-PEI/Cer, and we assessed its antitumor efficacy against HCC both *in vitro* and *in vivo* in combination with the chemotherapeutic drug sorafenib. We found that NGO-PEG-PEI significantly enhanced the cellular uptake of C6-ceramide. By investigating the mechanism of cellular delivery, we determined that the internalization of NGO-PEG-PEI/Cer progressed primarily via a clathrin-mediated mechanism. The combination of NGO-PEG-PEI/Cer and sorafenib exhibited synergy between these two drugs. Further work revealed that NGO-PEG-PEI/Cer may play a role in subverting multidrug resistance (MDR) in HCC cells by inactivating MDR and Akt signaling. NGO-PEG-PEI/Cer also significantly inhibited tumor growth and improved survival times *in vivo*, and the synergetic effect of NGO-PEG-PEI/Cer combined with sorafenib was also observed in drug-resistant HCC xenografts. In conclusion, our NGO-PEG-PEI nanocomposite is an effective nano-platform for loading C6-ceramide for therapeutic use in treating HCC, exhibiting high cancer cell killing potency in this tumor model. The NGO-PEG-PEI/Cer/sorafenib combination additionally represents a promising potential therapeutic strategy for the treatment of drug-resistant HCC.

## Introduction

Hepatocellular carcinoma (HCC) is the third most common cause of cancer-related death globally, accounting for approximately 80% of all types of primary liver cancer (Torre et al., 2015). Currently, HCC treatment is largely focused on a combination of surgery, chemotherapy, drug-targeted therapy and radiofrequency ablation as appropriate. These comprehensive treatments can significantly improve prognosis and prolong the life span of patients (Forner et al., 2012). Unfortunately, the majority of patients diagnosed with HCC are not eligible for surgery, leaving systemic therapy as the primary treatment option in those patients with advanced disease (Lencioni et al., 2016; Yu, 2016). Although conventional anticancer drugs have been used for the treatment of HCC, their high toxicity and relative non-specificity impede long-term application (Keating and Santoro, 2009). In addition, several clinical studies have demonstrated that conventional cytotoxic chemotherapy has low response rates and severe side effects (Thomas et al., 2008). Therefore, targeted drug delivery strategies and targeted therapeutics are the current key topics of research interest among those seeking to treat this deadly disease.

Ceramide, the simplest of the sphingolipids, is composed of a sphingosine base and amide-linked acyl chains varying in length from C14 to C26 (Ponnusamy et al., 2010). Ceramides have been reported to act as bioeffectors capable of mediating various cellular processes, including proliferation and apoptosis of cancer cells (Alphonse et al., 2013; Camacho et al., 2013). Work has revealed that ceramide has numerous effects on cell function, with the potential to induce cell growth arrest, senescence, apoptosis, and autophagy (Hannun and Obeid, 2008). Interestingly, ceramide can also mediate alternative pre-mRNA splicing, thereby enabling cells to express pro-apoptotic isoforms of bcl-x and caspase-9 (Chalfant et al., 2002; Massiello and Chalfant, 2006). Given these findings, ceramide has attracted tremendous attention in the field of cancer therapy as a potentially powerful tumor suppressor (Henry et al., 2013). C6-ceramide in particular has been widely used for the treatment of malignant tumors (Tagaram et al., 2011; Overbye et al., 2017). For example, Adiseshaiah et al. found that the synergistic combination therapy of nanoliposomal C6-ceramide and vinblastine is associated with a disruption of autophagy in HCC and colorectal cancer (Adiseshaiah et al., 2013). Tagaram et al. also demonstrated that ceramide induces p-AKT-dependent apoptosis in human HCC cells *in vitro* and suppresses xenograft tumor growth *in vivo* (Tagaram et al., 2011), exerting an inherent tumor-killing effect. However, ceramide is highly hydrophobic, which largely limits its application *in vivo*, necessitating the search for a suitable carrier for ceramide delivery that does not restrict its pharmacological effects.

Recently, nanoparticle therapy has been identified as a potential multi-modal approach to enhance therapeutic efficacy and reduce side effects associated with cancer treatment (Davis et al., 2008; Goncalves et al., 2013; Tao et al., 2017a, 2018). Nanoparticles are associated with a more targeted localization to tumors and an active mode cellular uptake, making it possible to achieve controlled-release drug delivery and specific gene transfection (Davis et al., 2008; Tao et al., 2013; Zhu et al., 2018). Graphene, a class of two-dimensional carbon nanomaterials with desirable physical and chemical properties, has attracted great interest in many different fields including biomedicine (Feng and Liu, 2011; Chen et al., 2012). Nano-graphene oxide (NGO) is reported to act as potential nano-platform for the delivery of anticancer chemotherapy drugs and genes (Zhang et al., 2010; Zhang L. et al., 2011). NGO was reported to be suitable for loading doxorubicin (DOX) at rates as high as 235% (the weight ratio of loaded drug to carriers) (de Melo-Diogo et al., 2018). However, the toxicology of NGO has been focused recently, which showed that its cellular toxicity *in vitro* is closely related to its surface functionalization (Hu et al., 2011). Zhang et al. developed DOX-loaded NGO-PEG (Polyethylene Glycol) as a strategy for chemo-photothermal synergistic therapy in one system, which significantly enhanced the therapeutic efficacy of cancer treatment *in vivo* and *in vitro* (Zhang W. et al., 2011).

NGO has great potential for use as delivery vehicles designed to enhance cancer treatment, So our collaborator developed PEG and PEI (Polyethylenimine) co-conjugated ultra-small nano-GO (NGO-PEG-PEI) as a novel gene delivery carrier, and found that it showed excellent stability against salts and serum (Feng et al., 2013). In the present study, we used these nanoparticles for loading C6-ceramide, and we found that this formulation allows C6-ceramide to travel through the bloodstream and target tumor cells via enhanced cellular permeability and retention, facilitating its potential clinical use as a novel therapeutic strategy. Additionally, through *in vitro* and *in vivo* studies we also investigated the antitumor efficacy and molecular mechanisms of NGO-PEG-PEI/Cer combined with other chemotherapy drugs in HCC.

## Materials and Methods

### Synthesis and Characterization of NGO-PEG-PEI/Cer

NGO-PEG-PEI was kindly provided by Dr. Kai Yang at the School of Radiation Medicine and Protection (SRMP) of Soochow University (Suzhou, China). Briefly, GO was obtained by oxidation of graphite following the modified Hummers method. Preparation of NGO-PEG-PEI was performed according to previous description (Feng et al., 2013). A mixture of GO solution (0.5 mg/ml) with 6-armed amine-terminated PEG (0.5 mg/ml) was under sonication for 5 min. Then EDC (0.5 mg/ml) was added, after another 5 min sonication, the mixture was stirred gently for 10 min at room temperature. The mixture was stirred for 6 h at room temperature following the second time addition of EDC (1 mg/ml) after being sonicated with PEI (2.5 mg/ml) for 5 min. After that, the mixture was washed with deionized water by 100 nm Milli-Q membrane filter (Millipore, Bedford, MA, United States) 3 times, and we obtained NGO-PEG-PEI re-suspended in water.

NBD C6-ceramide (6-((N-(7-Nitrobenz-2-Oxa-1,3-Diazol-4-yl)amino)hexanoyl)Sphingosine) (N1154, Thermo Fisher Scientific, MA, United States) solution with gradient concentration was prepared and its absorbance at 536 nm was measured. The standard curve was drawn according to different concentrations. Then C6-ceramide was mixed with a certain concentration of NGO-PEG-PEI solution in equal volume and oscillated overnight. After centrifuging for 30 min at 8000 rpm, the absorbance of supernatant was determined, and the concentration of free drug in supernatant was obtained according to the standard curve. Then NGO-PEG-PEI/Cer was prepared according to the maximum loading of C6-ceramide carried by NGO-PEG-PEI.

After loadinging the C6-Ceramide with NGO-PEG-PEI, PBS was added to make the final volume of 1.0 ml. The average size and zeta potential of the NGO-PEG-PEI/Cer complex were then measured with dynamic laser scattering (DLS) and a Zetasizer 3000HS particle analyzer (Malvern Instrument Inc., Worcestershire, United Kingdom), respectively. The sizes and zeta potential values were presented as the average values of three measurements.

### Cell Culture and Maintenance

The human HCC cell lines HepG2, HuH7, and PLC/PRF/5 were purchased from the Cell Bank of Type Culture Collection of Chinese Academy of Sciences (Shanghai, China). HuH7-SR is sorafenib-resistant HuH7 cell line, which was retained in our lab. All the cell lines were authenticated by short-tandem repeat profiling and cultured in Dulbecco’s Modified Eagle’s Medium (GIBCO, Carlsbad, CA, United States) supplemented with 10% heat inactivated fetal bovine serum (GIBCO). Cells were incubated in a 5% CO_2_ humidified incubator at 37°C.

### Cell Uptake of NGO-PEG-PEI/Cer

HCC cells were plated into 6-well plates at a density of 1 × 10^5^ cells/well and incubated with NGO-C6 in dulbecco’s modified eagle’s medium (DMEM) containing 10% fetal bovine serum. The cells were rinsed by PBS and then collected. The uptake ratio of NGO-PEG-PEI/Cer by HCC cells was measured by flow cytometry (Beckman, NJ, United States) ) using NBD labeled on NGO-PEG-PEI/Cer. The whole procedures were operated in dark place.

### Cell Viability Assay

For cell proliferation analysis, cells were dispensed in 96-well culture plates at a density of 5 × 103 cells/well. After attachment, cells were treated with Ceramide-C6, NGO-PEG-PEI/Cer, Sorafenib or combination therapy with NGO-PEG-PEI/Cer and Sorafenib at given concentration and time. The medium added with PBS was a blank control. Cell survival rate was evaluated by a standard 3-(4,5-dimethylthiazol-2-yl)-2,5-diphenyltetrazolium bromide (MTT) assay (Sigma, St. Louis, MO, United States), Medium was removed and fresh medium containing MTT (5 mg/ml) was added to each well. The cells were incubated at 37°C for 4 h, after draw off the supernatant of each well carefully and then an equal volume 150 μl of dimethyl sulfoxide (DMSO) was added to each well and mixed thoroughly on concentrating table for 10 min. The absorbance from the plates was read at 595 nm with Cytation 3 Multi-Mode Reader (BioTek, Vermont, United States).

### Cell Apoptosis Assay

Apoptosis staining Kit containing Annexin V- fluoresceine isothiocyanate (FITC)/Propidium iodide (PI) (KeyGene Biotech, Nanjing, China) was used to detect cell apoptosis according to the manufacture’s protocol. Cells were stained with 5 μl Annexin V-FITC and 5 μl PI after 48 h of treatment of NGO-PEG-PEI, C6 or NGO-PEG-PEI/Cer and then keep in dark at room temperature for 15 min. After that, these cells were analyzed by flow cytometer (Novo cyte 3130, ACEA Biosciences, Santiago, CA, United States).

### Western Blotting Analysis

Cells were harvested in lysis buffer (Beyotime, China) involving 1% Complete Mini-Protease Inhibitor Cocktail (Roche Diagnosis, Switzerland). Protein extractions were quantified using the bicinchoninic acid (BCA) kit (Thermo scientific, MA) and heated for 10 min at 100°C. Thirty microgram of protein was resolved in 12% SDS-PAGE and transferred to nitrocellulose membrane (Millipore, Germany). After blocked for 1 h at 37°C, the membranes were immunobloted with different antibodies overnight at 4°C. Antibodies against Caspase-8, Caspase-9, Caspase-3, recombinant poly ADP ribose polymerase (PARP), P-glycoprotein (P-gp), multidrug resistance 1 (MDR1), AKT serine/threonine kinase 1 (Akt), jun proto-oncogene (c-Jun), glyceraldehyde-3-phosphate dehydrogenase (GAPDH) were purchased from Abcam (Shanghai, China). Antibodies against Caspase-8 (1:1000), Caspase-9 (1:1000), Caspase-3 (1:1000), PARP (1:500), XIAP (1:1000), cIAP-1 (1:1000), cIAP-2 (1:1000), Survivin (1:1000), livin(1:1000), P-gp(1:500), and MDR1 (1:1000) were purchased from EMD Millipore Corporation (Billerica, MA, United States). Membranes were then washed with TBST and incubated with Horseradish Peroxidase (HRP)-conjugated goat anti-rabbit or anti-mouse antibody (1:5000) for 1 h at room temperature. Finally, blots were detected using ChemiDoc^TM^ MP Imaging System (Bio-Rad) with a SuperEnhancedchemiluminescence detection kit (Applygen, Beijing, China).

### Clonogenic Assay

The ability of the HCC cells to generate *in vitro* colonies was determined using clonogenic assay. Briefly, cells were incubated six-well plates at a concentration of 500 cells/well after treatment with NGO-PEG-PEI, C6 and NGO-PEG-PEI/Cer. The medium was regularly changed for 2 weeks until colony formation. Then the colonies were gently washed with phosphate buffer saline (PBS) after the supernatants removed, fixed with cold methanol for 20 min, and stained with crystal violet 0.1% in PBS at room temperature for 10 min followed by air-drying. Images were captured and the total number of colonies/well was counted.

### *In vivo* Antitumor Effect and Systemic Toxicity

Male Balb/c mice (6 weeks old) were obtained from Laboratory Animal Center of Zhejiang Chinese Medical University (Hangzhou, China). Tumor bearing mice were established by subcutaneous injection of 5 × 10^6^ HCC cells (HuH7 or HuH7-SR) in 200 μl PBS into the flank region of Balb/c mice. The dimension of tumors was monitored by digital calipers. Average tumor volume is about 90 mm^3^. Then the mice were randomized into 4 different treatment groups (7 mice per group): control group (PBS), NGO-PEG-PEI group, C6-ceramide group, NGO-PEG-PEI/Cer group. Then the mice bearing tumors in different groups were intravenous injected with PBS, NGO-PEG-PEI, C6-ceramide, NGO-PEG-PEI/Cer, respectively. After treatment, tumor volumes were tracked every 3 days by digital caliper measurements.

Control and nanocomposites-treated mice were sacrificed at 18 days after treatment. Major organs of those mice were collected, fixed in 4% formalin, conducted with paraffin embedded sections, stained with hematoxylin and eosin (H&E) and immuhistochemistry, and examined under a digital microscope.

This study was carried out in accordance with the recommendations of Laboratory Animal Center of Zhejiang Chinese Medical University, and the project was approved by the ethics committee of Zhejiang Provincial People’s Hospital.

### Statistical Analysis

The Statistical Package for the Social Sciences (version 13.0; SPSS Inc., Chicago, IL, United States) was used to perform all statistical analyses. Continuous data were analyzed using paired *t*-test or Wilcoxon rank test. Categorical data were analyzed using χ^2^ or Fisher’s exact test. Survival analysis was estimated by Kaplan–Meier method accompanying the log-rank test to calculate differences between the curves.

For all tests, *P*-values were obtained from two-tailed statistical tests and *p*-values less than 0.05 were considered statistically significant.

## Results

### Synthesis and Characterization of NGO-PEG-PEI/Cer Nanoparticles

Graphene oxide (GO) has been extensively explored in nanomedicine for its excellent physiochemical, electrical, and optical properties. In this study, polyethylene glycol (PEG) and polyethylenimine (PEI) were covalently conjugated to GO via amide bonds, yielding a physiologically stable dual-polymer-functionalized nano-GO conjugate (NGO-PEG-PEI) of ultra-small size. The synthesis and characterization of the NGO-PEG-PEI has been reported elsewhere (Feng et al., 2013). The NGO-PEG-PEI/Cer nanoparticles were generated via electrostatic interaction between the positively charged cationic NGO-PEG-PEI and the negatively charged C6-ceramide surface (Supplementary Figure 1). To determine optimal conditions for generating the NGO-PEG-PEI/Cer complex, the absorbance of the NGO-PEG-PEI/Cer were measured with various concentrations of the C6-Ceramide by UV Spectrophotometer. The size distribution and zeta potentials of the NGO-PEG-PEI/Cer complex were measured with various concentrations of the DA3 polymer by DLS and a zeta potential analyzer, respectively (Supplementary Figure 1).

**FIGURE 1:**
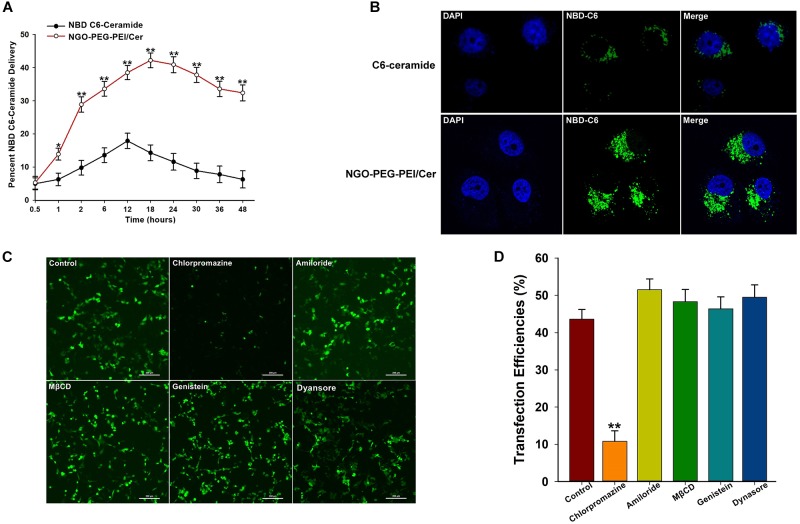
Characterization of NGO-PEG-PEI/Cer complex *in vitro*. **(A)** *In vitro* pharmacokinetics of C6-ceramide delivery by fluorescence spectrophotometry. NGO-PEG-PEI delivery of C6-ceramide resulted in a greater cellular accumulation of C6-ceramide as a function of time relative to naked C6-ceramide administration in the presence of 10% FBS. NGO-PEG-PEI were formulated with trace C6-ceramide to determine the kinetics of ceramide delivery to HCC cells. The total counts of NGO-PEG-PEI and naked C6-ceramide added to the cells were set at 100%. Error bars were based on triplicate samples, ^∗^*p* < 0.05, ^∗∗^*p* < 0.01 when comparing NGO-PEG-PEI/Cer accumulation with naked C6-ceramide accumulation. **(B)** Cell confocal microscopic images of NBD C6-ceramide (green) and cell nuclei (blue) were collected from HepG2 cells treated with NGO-PEG-PEI/Cer or C6-Ceramide. **(C)** To confirm the mechanisms of cellular uptake of the NGO-PEG-PEI/Cer complexes, cells were pre-treated for 30 min with inhibitors diluted in FBS-free media at the indicated concentrations. NGO-PEG-PEI/Cer complexes were then added in the absence or presence of inhibitors for an additional 2 h. Complexes were then removed and replaced with fresh 5% FBS-containing media and incubated for 24 h. Green fluorescence was observed by **(C)** fluorescence microscopy and **(D)** flow cytometry. Error bars were based on triplicate samples. ^∗∗^*P* < 0.01 versus control.

We incorporated trace amounts of C6-ceramide into NGO-PEG-PEI formulations to quantify the amount of NGO-PEG-PEI/Cer delivery compared with naked C6-ceramide administration. Pharmacokinetic studies revealed that NGO-PEG-PEI formulations delivered C6-ceramide more effectively and efficiently than did mock administration of C6-ceramide in the presence of 10% FBS (Figure 1A). NGO-PEG-PEI delivery resulted in a threefold increase in ceramide accumulation in HepG2 cells, with a maximal accumulation observed at approximately 18 h. Additionally, confocal microscopy demonstrated that NGO-PEG-PEI/Cer nanoparticles were present in the cytoplasm and enhanced transduction efficiency of C6-Ceramide in cancer cells (Figure 1B).

We next investigated the mechanism by which C6-ceramide is released or transferred from NGO-PEG-PEI vehicles into cellular membranes. To elucidate the mechanism of cellular delivery of NGO-PEG-PEI/Cer, we studied the effects of inhibitors of various endocytotic mechanisms, including chlorpromazine (clathrin inhibitor), amiloride (actin inhibitor), methyl-β-cyclodextrin (MβCD, caveolae inhibitor), genistein (PTK inhibitor), and dynasore (dynamin inhibitor). Cells without inhibitor pretreatment were studied as controls under the same experimental conditions. Relative green fluorescence levels were measured after transduction with NGO-PEG-PEI/Cer complexes, and the experimental results suggested that the cell entry process for NGO-PEG-PEI is via clathrin-mediated endocytosis, as chlorpromazine led to a 32.36% inhibition of internalization (Figure 1C,D).

### *In vitro* Antitumor Activity of NGO-PEG-PEI/Cer

**FIGURE 2:**
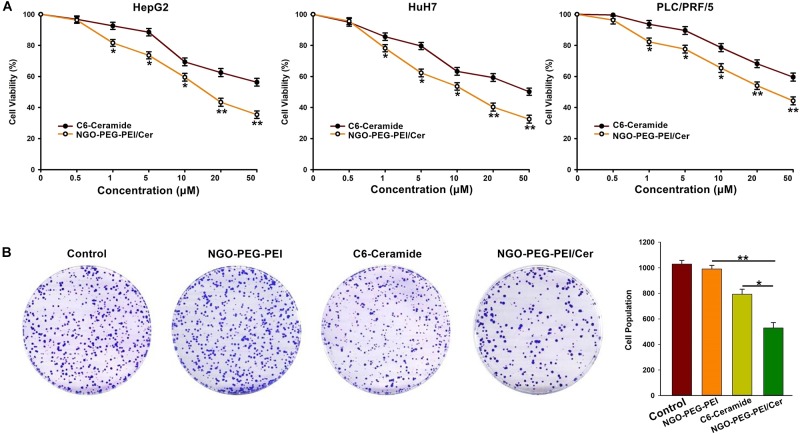
*In vitro* anti-tumor activity of NGO-PEG-PEI/Cer. **(A)** Cells were seeded in 96-well plates and infected with NGO-PEG-PEI/CER or C6-ceramide at a series of concentrations. Cell viability was determined by MTT cell proliferation assays at 48 h post-infection. The results were presented as mean ± SD of three separate experiments. **(B)** Clonogenic formation of HepG2 cells treated by NGO-PEG-PEI/Cer. ^∗^*P* < 0.05, ^∗∗^*P* < 0.01.

A 3-(4,5-dimethylthiazol-2-yl)-2,5-diphenyltetrazolium bromide (MTT) assay was performed 48 h post-infection with NGO-PEG-PEI/Cer to evaluate the cytotoxicity of the nanocomposites in the HepG2, HuH7 or PLC/PRF/5 cell lines in the presence of 10% FBS. The results indicated a significantly higher inhibition of cell growth in cells treated with NGO-PEG-PEI/Cer relative to those treated with C6-ceramide in a dose-dependent fashion (Figure 2A).

To further confirm the inhibitory effect of NGO-PEG-PEI/Cer on the proliferation of HCC cells, we conducted a clone formation assay and found that NGO-PEG-PEI/Cer could effectively inhibit the proliferation of HCC cells (Figure 2B).

### NGO-PEG-PEI/Cer Treatment Induces Apoptosis *in vitro*

**FIGURE 3:**
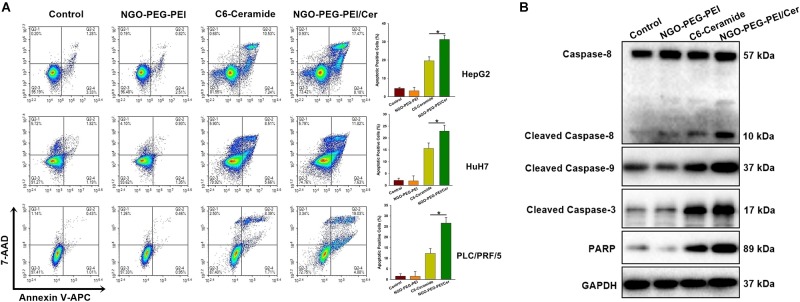
NGO-PEG-PEI/CER induced apoptosis in HCC cells *in vitro*. **(A)** Apoptosis was analyzed via Annexin V-FITC/PI double staining. HepG2, HuH7, or PLC/PRF/5 cells were infected with NGO-PEG-PEI/Cer (10 μM), C6-ceramide (10 μM), or NGO-PEG-PEI (100 μg/ml) for 24 h. Florescence was then analyzed by flow cytometry. Data are presented as mean ± SD of three separate experiments. **(B)** HepG2 cells were infected with NGO-PEG-PEI/Cer (10 μM), C6-ceramide (10 μM), or NGO-PEG-PEI (100 μg/ml) for 24 h. Whole cell extracts were prepared and immunoblotted to detect caspase pathway activation. GAPDH was used as a loading control. ^∗^*P* < 0.05.

To address the underlying mechanism by which NGO-PEG-PEI/Cer induces cytotoxicity, we evaluated NGO-PEG-PEI/Cer associated apoptosis *in vitro* by flow cytometry. We observed significant increases in apoptosis in the HepG2, HuH7 or PLC/PRF/5 cell lines treated with NGO-PEG-PEI/Cer relative to those threated with C6-ceramide, NGO-PEG-PEI or PBS (Figure 3A).

We further evaluated apoptosis by assessing the expression of apoptosis-related proteins in HepG2 cells at 48 h post-infection using Western blotting analysis. The results indicated a significant increase in the activation of caspases 3, 8, and 9, and increased PARP cleavage in NGO-PEG-PEI/Cer-treated cells (Figure 3B). Taken together, these findings indicated that NGO-PEG-PEI/Cer effectively induced apoptosis via caspase activation.

### Combined Treatment With Sorafenib and NGO-PEG-PEI/Cer Results in Synergistic Efficacy

**FIGURE 4:**
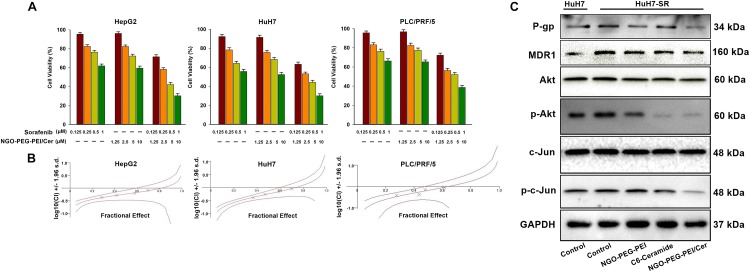
NGO-PEG-PEI/Cer enhances sorafenib-mediated growth inhibition in HCC cells. **(A)** Cells were treated with NGO-PEG-PEI/Cer and/or sorafenib for 48 h, and cell viability was then determined by MTT assay. Data are presented as mean ± SD of three separate experiments. **(B)** The potential synergistic effect of sorafenib combined with NGO-PEG-PEI/Cer on HCC cells was assessed by Chou–Talalay Combination Index (CI) analysis using the CalcuSyn software. The middle curve line indicates the simulated combination index values, which are expressed as the log10 (CI) ± 1.96 SD, encircled by two lines of algebraic evaluation of the 95% confidence intervals. The log10 (CI) values attained at given fractional affects represent an antagonism between the treatments when >0, an additive efficiency when equal to 0 and a synergism when <0. This was quantified by CIN analysis and expressed as CIN versus fraction affected. Where calculable, 95% confidence intervals are shown. **(C)** The sorafenib-resistant HuH7 cell line (HuH7-SR) was used to illuminate the mechanism by which NGO-PEG-PEI/Cer may influence sorafenib-resistant HCC. NGO-C6(10 μM), C6-ceramide (10 μM), or NGO (100 μg/ml) was used to treat HuH7-SR cells. Uninfected cells served as control. Forty eight hours later, whole cell extracts were prepared and immunoblotted. GAPDH was used as a loading control.

To determine whether NGO-PEG-PEI/Cer enhances the cytotoxic effect of sorafenib, we analyzed the viability of HCC cells after co-treatment with NGO-PEG-PEI/Cer and sorafenib. The HepG2, HuH7, and PLC/PRF/5 cells were treated with sorafenib (0.125, 0.25, 0.5, or 1 μM) with or without NGO-PEG-PEI/Cer (1.25, 2.5, 5, or 10 μM). The combination of NGO-PEG-PEI/Cer with sorafenib significantly inhibited cell growth as compared with treatment with sorafenib or NGO-PEG-PEI/Cer alone (Figure 4A). Next, the synergistic effects of sorafenib combined with NGO-PEG-PEI/Cer on HCC cells were quantified by combination index (CIN) analysis and expressed as CIN versus fraction affected in Figure 4B. These results revealed that the combination of sorafenib and NGO-PEG-PEI/Cer has a synergistic tumor killing effect.

These results raised the question of the mechanism by which NGO-PEG-PEI/Cer influences sorafenib-resistant HCC cell lines. Therefore, we generated a sorafenib-resistant HuH7 cell line (HuH7-SR) and assessed the expression of multidrug resistance-related proteins or Akt, phospho-Akt, c-Jun, phospho-c-Jun of these HuH7-SR cells treated with NGO-PEG-PEI/Cer, C6-ceramide, or NGO-PEG-PEI by Western blot analysis in the presence of 10% FBS. Compared with the sensitive HuH7 cells, HuH7-SR cells exhibited markedly elevated levels of multidrug resistance-related proteins (P-gp and MDR1) and Akt, phospho-Akt, c-Jun, phospho-c-Jun. Our results further demonstrate that NGO-PEG-PEI/Cer has the capacity to reduce levels of multidrug resistance-related proteins and Akt, phospho-Akt, c-Jun, phospho-c-Jun (Figure 4C). Taken together, these findings indicated a synergistic repressive effect of the combination of sorafenib and NGO-PEG-PEI/Cer treatment on HCC cell proliferation.

### Enhanced Cytotoxic Effect of Co-treatment With Sorafenib and NGO-PEG-PEI/Cer *in vivo*

**FIGURE 5:**
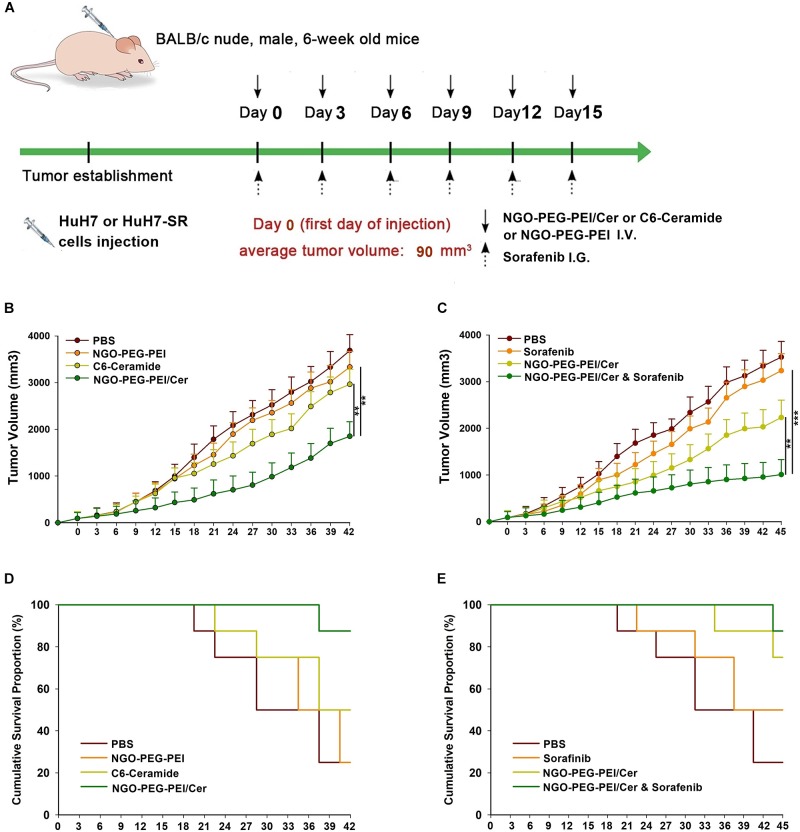
Antitumor efficacy of NGO-PEG-PEI/Cer and synergistic effects of sorafenib and NGO-PEG-PEI/Cer *in vivo*. **(A)** Schematic of experimental timeline. BALB/c athymic nude mice implanted with HuH7 or HuH7-SR tumor xenografts were intravenously injected with PBS (100 μl), C6-ceramide 5 mg/kg), or NGO-PEG-PEI/Cer (5 mg/kg) every 3 days for a total of 6 times, or intragastric administration with a single dose of sorafenib (10 mg/kg) every 3 days for 6 times, or with a combination of sorafenib and NGO-PEG-PEI/Cer at the same conditions as above. **(B,C)** Tumor volumes were measured at different times after treatment. Data are presented as mean ± standard error (*n* = 6). ^∗^*P* < 0.05; ^∗∗^*P* < 0.01; and ^∗∗∗^*P* < 0.001, one-way analysis of variance (ANOVA) and multiple comparisons. **(D,E)** Images show the inhibitory effects of each treatment on tumor growth based on survival.

We developed two hepatoma carcinoma tumor xenograft mouse models using the HuH7 cells and HuH7-SR cells in BALB/c athymic nude mice to evaluate the effects of NGO-PEG-PEI/Cer treatment or co-treatment with sorafenib and NGO-PEG-PEI/Cer *in vivo* (Figure 5A). Anti-tumor efficacy was evaluated by plotting tumor growth curves over a 42 or 45 days observation period. The mean tumor volume was significantly decreased in mice injected with sorafenib, NGO-PEG-PEI/Cer, and the combination therapy relative with those injected with PBS (Figure 5B,C). Furthermore, co-treatment of sensitive HuH7 and HuH7-SR cells with sorafenib and NGO-PEG-PEI/Cer was more effective than sorafenib (*P* = 0.001 and 0.002, respectively) and NGO-PEG-PEI/Cer alone (*P* = 0.001 and 0.003, respectively). Co-treatment with sorafenib and NGO-PEG-PEI/Cer was also associated with a higher survival rate than treatment with PBS, sorafenib, or NGO-PEG-PEI/Cer (Figure 5D,E).

**FIGURE 6:**
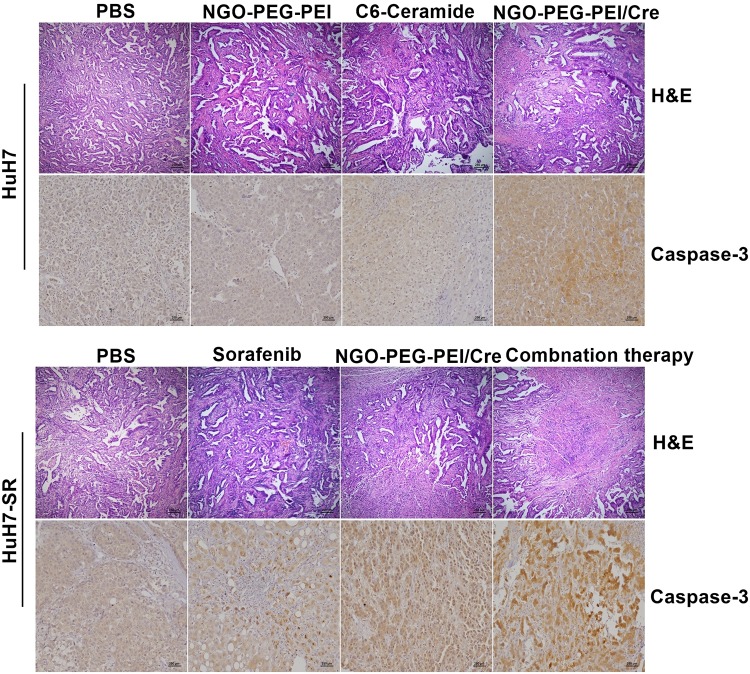
Histopathological analysis. Subcutaneous HuH7 or HuH7-SR tumors were collected 18 days after injection, and sections were analyzed by the indicated methods. H&E staining showed that tumor tissues treated with the NGO-PEG-PEI/Cer or combination of sorafenib and NGO-PEG-PEI/Cer exhibited the greatest amount of cell death. An immunohistochemical analysis demonstrated there was strong expression of caspase-3 in xenografts treated with NGO-PEG-PEI/Cer as well as in those from the combined therapy group.

The tumor histopathological changes were further evaluated by hematoxylin and eosin (H&E) staining and immunohistochemistry (IHC). The combined treatment with sorafenib and NGO-PEG-PEI/Cer resulted in greater cytotoxicity than either single treatment as evidenced by H&E staining. Moreover, very high Caspase-3 expression was evident in the tumor tissues from mice which received the combined treatment based on IHC staining with an anti-Caspase-3 antibody (Figure 6). Results from this experiment showed significantly higher rates of apoptosis in the combination treatment group when compared with either individual treatment (Figure 6).

## Discussion

Ceramide is reported to modulate cell death, cycle arrest, metastasis, stress responses, and pro-inflammatory responses in cancer cells (Saddoughi and Ogretmen, 2013; Kitatani et al., 2016), especially in HCC (Morales et al., 2007). Despite its key role in regulating tumor cell growth and death, its cell impermeability and its tendency to undergo precipitation in aqueous solutions have limited the use of ceramide as a therapeutic agent.

With the development of nanotechnology, application of nanomedicine in drug delivery becomes an area of rapid growth and advancement given its significant ability to enhance therapeutic efficacy, minimize side effects of drugs, and enhance drug bioavailability *in vivo* (Chen et al., 2016; Taghdisi et al., 2016; Tao et al., 2016, 2017b). Therefore, many nanomaterials – namely liposomes, micelles, dendrimers, carbon nanotubes, polymers, inorganic metallic nanolayers, and graphene oxides – have been explored in efforts to design nanocarriers for different drugs (Elgadir et al., 2015; Pattni et al., 2015). Recently, NGO has been explored in the field of biomedicine, which was found to be promising for drug delivery (Huang et al., 2012; Jayakumar et al., 2012). It is attractive in part due to its ease of synthesis, controlled particle size, and high surface area for drug loading. Although NGO without additional surface coatings appears to exhibit dose-dependent toxicity, well functionalized NGO with biocompatible coatings such as PEG has been found to reduce obvious toxicity (Yang et al., 2013a,b). Other studies have explored the use of NGO for DNA plasmid and siRNA delivery after being functionalized by PEI (Feng et al., 2011; Zhang L. et al., 2011). Our collaborator also found that PEG and PEI functionalized NGO may enhance gene delivery (Feng et al., 2013). In the present study, we used NGO-PEG-PEI to carry C6, and we determined that the maximum drug loading capacity of 100 μg/ml NGO-PEG-PEI was 56 μM C6-ceramide. Next, in order to verify the enhanced intracellular trafficking of NGO-PEG-PEI/Cer, we assessed the cell uptake of NBD labeled NGO-PEG-PEI/Cer. Our results revealed that NGO-PEG-PEI significantly enhanced the cellular uptake of C6 and resulted in a threefold increase in ceramide accumulation with a maximal accumulation observed after approximately 18 h, which revealed that NGO-PEG-PEI formulations delivered C6-ceramide more effectively and efficiently. The mechanism of cellular delivery was also explored, established that this intracellular internalization of NGO-PEG-PEI/Cer progressed mainly via a clathrin-mediated mechanism.

C6-ceramide was cytotoxic and anti-proliferative when employed against a panel of human melanoma cells (Jiang et al., 2016), as well as against cervical cancer and colon cancer cells (Overbye et al., 2017). Nanoliposomal C6-ceramide has also been reported to induce cell apoptosis of HCC cells *in vitro*, concomitant with an accumulation of cells in the G2 phase of the cell cycle (Tagaram et al., 2011). In the present study, cell viability and toxicity were also detected after treatment with NGO-PEG-PEI/Cer, and our results showed that NGO-PEG-PEI/Cer exhibited high tumor cell killing potency as it was capable of both reducing HCC cell proliferation and increasing apoptosis. These findings thus suggest that NGO-PEG-PEI/Cer may induce potent, specific antitumor cytotoxicity.

Sorafenib, which is the first multi-target, multi-kinase inhibitor to be developed, is systematically used in the treatment of advanced liver cancer and has been proven to be effective (Wilhelm et al., 2006; Cheng et al., 2009). However, many patients do not respond to sorafenib, or they develop drug resistance after several months of sorafenib treatment. Therefore, it is urgent that enhancers or synergistic agents be identified for combination use with sorafenib to improve the clinical treatment of HCC. In our study, NGO-PEG-PEI/Cer combined with sorafenib was utilized for treating HCC, and the CI50 was determined to establish a synergistic effect. Our results showed that combination treatment with both drugs showed clear synergism effects superior to single drug treatment. NGO-PEG-PEI/Cer combined with sorafenib displayed an overall CI50 value <1, confirming the synergy between these two drugs. Further work revealed that the NGO-PEG-PEI/Cer/sorafenib combination may exert anti-multidrug resistance (anti-MDR) activities in HCC cells by significant inactivation of MDR and Akt signaling. These results illustrated that NGO-PEG-PEI/Cer combined with sorafenib can mediate an efficient synergistic therapeutic effect for antitumor therapy in drug-resistant HCC.

To evaluate the effects of NGO-PEG-PEI-C6 on HCC tumor-bearing mouse xenografts, survival time and tumor volume were evaluated, revealing that NGO-PEG-PEI/Cer significantly delayed tumor growth and improved survival times. Li et al. previously found that injection of nanoliposome-loaded C6-ceramide slowed tumor growth by reducing proliferation and increasing apoptosis in HCC (Li et al., 2018), suggesting that delivery system of ceramide via nanoparticle may be an effective strategy for the treatment of human HCC *in vitro* and *in vivo*. The synergetic effects of NGO-PEG-PEI/Cer combined with sorafenib were also evident in our mouse model, with evidence of tumor growth inhibition and improved overall survival improvement in mice receiving the combination regimen. These findings suggest that the NGO-PEG-PEI/Cer/sorafenib combination represents a potential therapeutic strategy for the treatment of drug-resistant HCC *in vivo*.

In summary, using nanotechnology-based advances in drug delivery, we utilized NGO-PEG-PEI nanocomposites as an effective nano-platform for loading C6-ceramide for therapeutic treatment of HCC. This formulated composite exhibited excellent anti-cancer efficacy *in vitro* and in *vivo*, facilitating its potential clinical use. Furthermore, combined treatment with NGO-PEG-PEI/Cer and sorafenib achieved a superior therapeutic effect in drug-resistant HCC, suggesting that NGO-PEG-PEI/Cer has great potential to treat drug-resistant HCC when used as a synergistic agent in combination with sorafenib.

## Author Contributions

X-ZM conceived and designed the experiments. S-BW and Y-YM carried out the majority of experiments and drafted the manuscript. X-YC analyzed the results and revised the manuscript. Y-YZ collected and analyzed the data. All authors read and approved the final manuscript.

## Conflict of Interest Statement

The authors declare that the research was conducted in the absence of any commercial or financial relationships that could be construed as a potential conflict of interest.

## Supplementary Material

FIGURE S1 Synthesis and characterization of NGO-PEG-PEI/Cer. **(A)** The zeta potential value of the NGO-PEG-PEI were measured. **(B)** A schematic illustration showing the synthesis of NGO-PEG-PEI conjugate and the preparation of NGO-PEG-PEI/Cer complex. **(C)** The absorbance of the NGO-PEG-PEI/Cer were measured with various concentrations of the C6-Ceramide by UV Spectrophotometer. **(D)** The average size (nm) of the NGO-PEG-PEI/Cer complex was measured.
