# Supplementary figures and images for: Ceramide-Graphene Oxide Nanoparticles Enhance Cytotoxicity and Decrease HCC Xenograft Development: A Novel Approach for Targeted Cancer Therapy
Source: Front Pharmacol. 2019 Feb 8;10:69. doi: 10.3389/fphar.2019.00069 (PMC6376252; doi:10.3389/fphar.2019.00069)

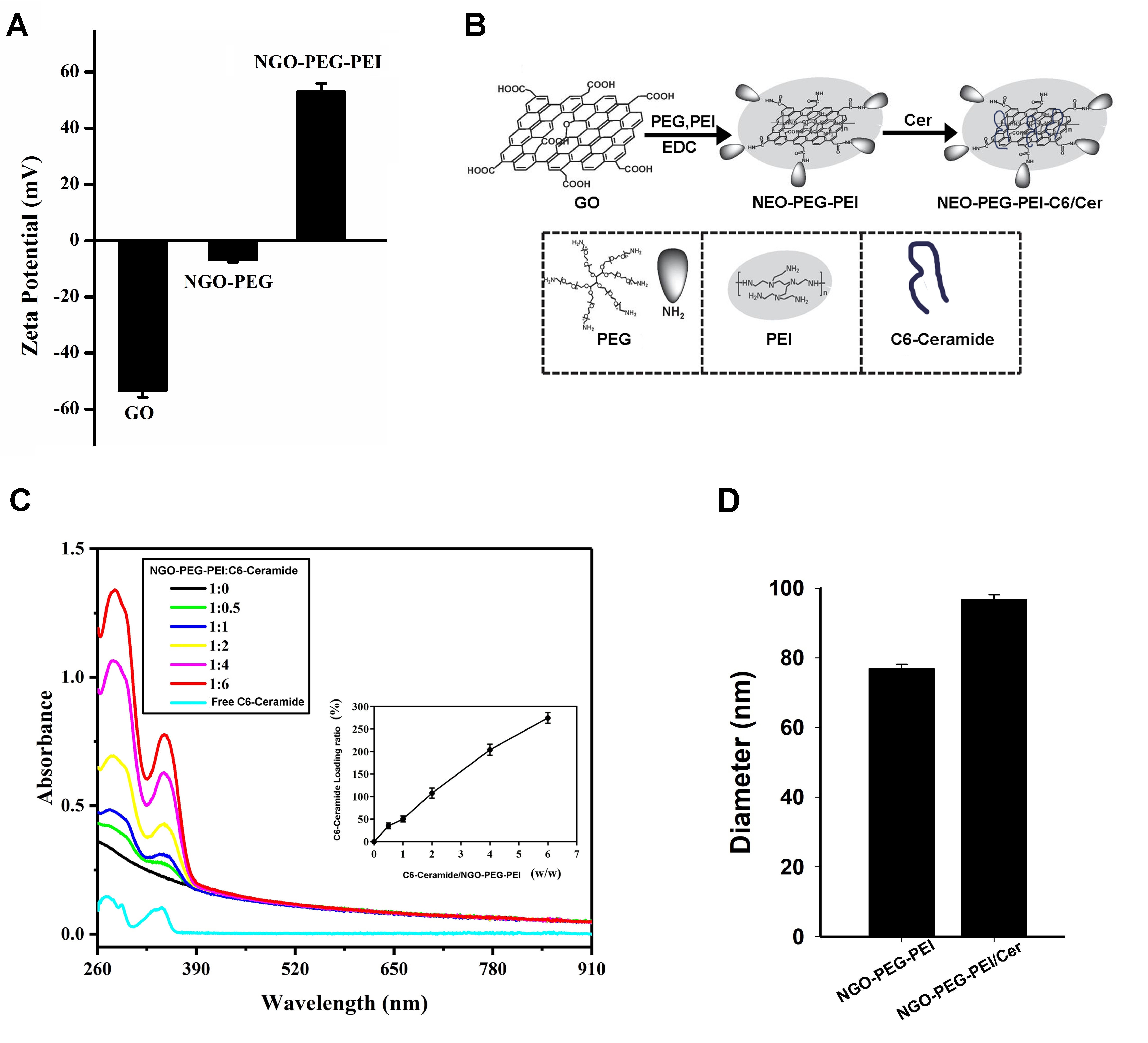

Supplement: FIGURE S1 — Synthesis and characterization of NGO-PEG-PEI/Cer. (A) The zeta potential value of the NGO-PEG-PEI were measured. (B) A schematic illustration showing the synthesis of NGO-PEG-PEI conjugate and the preparation of NGO-PEG-PEI/Cer complex. (C) The absorbance of the NGO-PEG-PEI/Cer were measured with various concentrations of the C6-Ceramide by UV Spectrophotometer. (D) The average size (nm) of the NGO-PEG-PEI/Cer complex was measured. [file Image_1.JPEG]
